# Birth preparedness and complication readiness among pregnant women admitted in a rural hospital in Rwanda

**DOI:** 10.1186/s12884-018-1818-x

**Published:** 2018-05-30

**Authors:** Patrick Smeele, Richard Kalisa, Marianne van Elteren, Jos van Roosmalen, Thomas van den Akker

**Affiliations:** 1 Department of Medical Humanities, VU University Medical Center, Amsterdam, the Netherlands; 2 Department of Obstetrics and Gynecology, Ruhengeri Hospital, Musanze, Rwanda; 3 Athena Institute, VU University Medical Center, Amsterdam, the Netherlands; 4 Department of Obstetrics, Leiden University Medical Center, Leiden, the Netherlands

**Keywords:** Birth preparedness, Complication readiness, Obstetrics, High-risk pregnancy, Health promotion, Rwanda

## Abstract

**Background:**

With an aim to prevent adverse pregnancy outcomes, ‘birth preparedness and complication readiness’ (BP/CR) promotes timely access to skilled maternal and neonatal services. Objective of this study was to assess implementation of BP/CR among pregnant women admitted with obstetric emergencies in rural Rwanda.

**Methods:**

A cross-sectional study among pregnant women who were referred to Ruhengeri hospital between July and November 2015. The ‘Safe Motherhood questionnaire’ as developed by Jhpiego’s Maternal and Neonatal Health Program was used to collect data. Women were asked to mention key danger signs and respond as to whether they had identified: (A) skilled birth attendant, (B) location to give birth, (C) mode of transport, (D) money to cover health care expenditure. Women who answered ‘yes’ to three or four items were labeled ‘well prepared’. Multivariate logistic regression analysis was conducted to compare the ‘well prepared’ and ‘less prepared’.

**Results:**

With regard to complication readiness, out of 350 women, 296 (84.6%), 271 (77.4%) and 288 (82.3%) could mention at least one key danger sign during pregnancy, labor and postpartum respectively, but only 23 (6.6%) could mention three or more key danger signs during all three periods. With regard to birth preparedness, 46 (13.1%) women had identified a skilled birth attendant, 68 (19.4%) birth location, 76 (21.7%) mode of transport, and 306 (87.4%) had saved money for health care costs. Seventy-eight women (22.3%) were ‘well prepared’, associated factors being first time pregnancy (adjusted Odds Ratio (aOR) = 3.2; 95% CI; 1.2–5.8), knowledge of at least two danger signs (aOR = 2.8; 95% CI; 1.7–3.9) and having been assisted by a community health worker at the antenatal clinic (aOR = 2.2, 95% CI; 1.3–3.7).

**Conclusion:**

Knowledge of obstetric danger signs was suboptimal and birth preparedness low. We recommend review of practices regarding health promotion in antenatal care, taking care not to exclude multiparous women from messages related to birth preparedness, and do promote use of community health workers to enhance effectiveness of BP/CR.

**Electronic supplementary material:**

The online version of this article (10.1186/s12884-018-1818-x) contains supplementary material, which is available to authorized users.

## Background

Maternal mortality remains a major global concern, especially in sub-Saharan Africa where the maternal mortality ratio, although declining, is still high [1–3]. One of the reasons is lack of Birth Preparedness and Complication Readiness (BP/CR), which is recognized as a key component of safe motherhood programs around the world [4, 5].

BP/CR is a comprehensive package to promote timely access to skilled maternal and neonatal health services. It also promotes active preparation and decision making for birth among pregnant women and their families [5–7]. A birth plan includes identifying a skilled birth attendant and location of the closest appropriate care facility, saving funds for birth-related and emergency expenses, arranging transport to a health facility for birth and obstetric emergencies and identification of compatible blood donors in case of need [5]. The latter criterion does not apply in Rwanda, where centralized blood banks taking blood from voluntary donors are in place [8].

Whilst BP/CR has been associated with reduced maternal and neonatal mortality [9], improved preventive behaviors [10–12], increased knowledge of danger signs [13–15] and more frequent seeking of professional care during emergencies [11, 16, 17], previous studies have shown low rates of BP/CR among women in Uganda [18], Ethiopia [4, 14, 15] and Burkina Faso [19]. The rate of BP/CR among women in Rwanda is unknown.

In 2003, Rwanda adopted BP/CR as part of ‘focused antenatal care’ to increase access to skilled birth attendance [20, 21]. Part of this strategy is that health workers explain women the obstetric danger signs that may occur during pregnancy, childbirth and the postpartum period as well as methods to prevent mother-to-child transmission of HIV [20]. The introduction of focused antenatal care may have contributed to the reduction of the maternal mortality ratio by roughly two-thirds from 750 in 2005 to 210 per 100,000 live births in 2015 and to the increased skilled birth attendance rate from 28 to 91% [22, 23].

This study aimed to assess practices around and factors associated with BP/CR among pregnant women admitted with obstetric emergencies in a rural Rwandan hospital.

## Methods

### Design

This was a cross-sectional study among pregnant women who were referred for obstetric emergencies to Ruhengeri hospital, Musanze district, Rwanda, between July and November 2015.

### Setting

According to the Population Census, Musanze district had a population of 368,267 inhabitants with a total fertility rate of 4.6 births per woman in 2012. Health insurance coverage was 85.1%, and 65.3% of women who gave birth with assistance from a skilled birth attendant. Uptake of postnatal care by skilled personnel was 4.5% [24]. Health promotion and counseling as part of BP/CR are provided by community health workers in addition to other facility-based professionals. Community health workers sometimes escort laboring women to health facilities.

Ruhengeri hospital acts as a provincial referral hospital for women with high-risk pregnancies and referrals from health centers and other district hospitals in the northern province. Medical services offered are covered by community-based health insurance (‘Mutuelle de Santé’) at contribution of an annual fee of RWF 3000 (US$4.5), with a 10% surcharge for each episode of illness. In case of shortages of supplies, patients are requested to procure missing items from private pharmacies. During the study period, medical staff consisted of one specialist obstetrician, four medical officers, two intern doctors and 18 midwives.

### Data collection

The study included all pregnant women who were referred to the maternity ward who consented to participation, using the consent form given in Additional file 1. Participants were followed up to discharge or death.

Two trained research assistants identified possible participants while the principal investigator verified suitability for study inclusion. The ‘Safe Motherhood questionnaire’ developed by the Maternal Neonatal Program of JHPIEGO, an affiliate of John Hopkins University [5] was used, and adapted to the local context to include a question regarding purchase of birth materials as a common birth preparedness practice (Additional file 1). The expert translator translated it from the English version to the local language (Kinyarwanda), and then another translator translated this text back into English to check whether the original meaning was still present.

The questionnaire pertained to socio-demographic variables such as age, residence, religion, education level, marital and employment status, and other variables with regard to antenatal care (including type of advice received and type of health worker seen), obstetric history, reasons for referral. With regard to knowledge of obstetric danger signs, we assessed whether a woman, when prompted, could mention danger signs and symptoms such as vaginal bleeding, fits, swelling of face or limbs, fever, loss of consciousness, headache, abdominal pain, prolonged labor and retained placenta.

Lastly, four ‘BP/CR questions’ verified whether the woman had taken one of the following four steps: A) identification of a skilled birth attendant, B) identification of the location of the closest appropriate care facility, C) identification of a means of transport to that facility, D) saving money for hospital costs/birth materials. Women answering ‘yes’ to at least three of these four BP/CR questions were labeled ‘well prepared’. Remaining women were labeled ‘less prepared’. We also assessed whether mentioning of at least two danger signs during pregnancy, childbirth or postpartum was associated with being well prepared.

### Data analysis

Data were entered, coded, cleaned and analyzed using SPSS for Windows Version 18.0. After the initial descriptive analysis, bivariate analysis was done to test for associations between the dependent variable BP/CR and independent variables using Pearson’s chi square or Fischer’s exact test. Factors that were found to have *p*-values below 0.2 in the bivariate analysis were entered into multivariable logistic regression model to compare women who were well prepared with those who were less prepared.

## Results

Of all 350 women who were interviewed, mean age was 27.7 years. Characteristics are shown in Table 1.

**Table 1:** Socio-demographic and obstetric characteristics

Characteristics	Number (*n*)	Percent (%)
Age (Years) (Mean ± SD; 27.7 ± 6.0)		
< 20	35	10.0
21- 29	188	53.7
> 30	127	36.3
Marital status		
Married	327	93.4
Not currently married^a^	23	6.6
Residence (district)		
Musanze	267	76.3
Others^b^	83	23.7
Education		
None	114	32.6
Primary	193	55.1
Secondary and Above	43	12.3
Occupation		
Housewife	195	55.7
Own business/private employee	98	28.0
Government/salaried employee	57	16.3
Religion		
Christianity	318	90.9
Islam	32	9.1
Parity (Mean ± SD; 2.6 ± 1.9)		
1	123	35.1
2–4	176	50.3
> 5	51	14.6
Prior stillbirth		
No	290	82.9
Yes	60	17.1
Travel time to health facility		
< 1 h	215	61.4
≥ 1 h	135	38.6

Mean ± Standard Deviation

^a^Single, divorced and widowed

Other^b^ Nyabihu/Rubavu/Burera/Gakeke

All respondents had attended ANC at least once during this pregnancy; 131 women (37.4%) had completed the recommended four or more antenatal visits. Mean antenatal visits were 2.9 ± 0.9. Almost two out of three women (59.4%) had received education on the importance of knowing danger signs, knowing where to go in case of complications (73.1%) and where to give birth (76.3%), identifying transport (67.1%), identifying a skilled birth attendant (17.7%) and saving money (76.9%) (Table 2).

**Table 2:** Antenatal care uptake and advice given

Characteristics	Number	Percent
Antenatal attendance (Mean ± SD; 2.9 ± 0.9)		
≥ 4	131	37.4
2-3	185	52.9
1	34	9.7
Gestational age at first antenatal visit		
1st trimester	267	76.3
2nd trimester	60	17.1
3rd trimester	23	6.6
Personnel checked		
Health professional	147	41.7
Community health workers	203	58.3
Advice on danger signs during pregnancy, childbirth, or postpartum		
Yes	208	59.4
No	142	40.6
Advise on where to go if danger signs happen		
Yes	256	73.1
No	94	26.9
Advise on identifying health facility		
Yes	267	76.3
No	83	23.7
Advise on arrangement for transport		
Yes	235	67.1
No	115	32.9
Advise on saving money for delivery or emergency		
Yes	269	76.9
No	81	23.1
Advise on identifying skilled birth attendant		
Yes	62	17.7
No	288	82.3

Regarding knowledge of key danger signs, vaginal bleeding was the most frequently mentioned complication by women during pregnancy (61.1%), labor/birth (73.1%) and postpartum (58%) (Table 3). Prolonged labor, which is one of the leading causes of maternal morbidity, was reported by only 13.7%. Most women knew at least one key danger sign during pregnancy (*n* = 296; 84.6%), labor/birth (*n* = 271; 77.4%) and postpartum (*n* = 288; 82.3%). Only 23 women (6.6%) had knowledge of three or more key danger signs during the three periods.

**Table 3:** Women’s awareness of obstetric danger signs during pregnancy, birth and postpartum

Obstetric danger signs	Awareness					
	Pregnancy		Labor/Childbirth		Postpartum	
	*n*	%	*n*	%	*n*	%
Vaginal bleeding	214	61.1	256	73.1	203	58.0
Fits of pregnancy	15	4.3	11	3.1	2	0.6
Swelling of face/lower limbs	52	14.9			98	28.0
High grade fever	20	5.7	13	3.7	18	5.1
Loss of consciousness	41	11.7	3	0.9	29	8.3
Severe headache	39	11.1	19	5.4	67	19.1
Dizziness/blurred vision	31	8.9			22	6.3
Severe abdominal pain	50	14.3			46	13.1
Baby does not move	22	6.3				
Difficulty in breathing	14	4.0			9	2.6
Severe weakness	67	19.1			41	11.7
Water breaks without labor	88	25.1				
Prolonged labor			48	13.7		
Retained placenta			125	35.7		
Foul smelling vaginal discharge					30	8.6
Do not know any of the above	54	15.4	79	22.6	62	17.7

In practice, 46 women (13.1%) had identified a skilled birth attendant, 68 (19.4%) a facility to give birth, and 76 (21.7%) a means of transportation. Most women (*n* = 306; 87.4%) had saved money for hospital costs/birth materials (Table 4). About one in five women (*n* = 78; 22.3%) were considered ‘well prepared’ in terms of BP/CR.

**Table 4:** Birth preparedness among pregnant women

Level of birth preparedness	Number	Percent
Identified health facility		
Yes	68	19.4
No	282	80.6
Arranged for transport		
Yes	76	21.7
No	274	78.3
Saved money		
Yes	306	87.4
No	44	12.6
Identified skilled birth attendant		
Yes	46	13.1
No	304	86.9
Number of steps taken		
0	81	23.1
1	129	36.9
2	62	17.7
3	66	18.9
4	12	3.4
At least 3 steps taken	78	22.3

The adjusted multivariate model showed that significant predictors for being well prepared were first time pregnancy (adjusted odds ratio (aOR) = 3.2; 95% CI 1.2–5.8), knowledge of at least two danger signs during pregnancy (aOR = 2.8; 95% CI 1.7–3.9) and having seen a community health worker (aOR = 2.2, 95% CI 1.3–3.7) (Table 5).

**Table 5:** Characteristics of well-prepared^a^ women versus those less-prepared

Characteristics	Birth preparedness		COR (95% CI)	^b^aOR (95% CI)
	Well ^a^(*n* = 78)	Less (*n* = 272)		
Age (Years)				
< 25	41 (52.6)	167 (61.4)	0.9 (0.4-2.0)	0.6 (0.5-1.4)
≥ 25	37 (47.4)	105 (38.6)	1.0	
Marital status				
Married	70 (89.7)	257 (94.5)	1.0	
Not currently married^c^	8 (10.3)	15 (5.5)	2.0 (0.8-4.8)	1.2 (0.3-4.2)
Occupation				
Irregular income	66 (84.6)	227 (83.5)	1.0	
Regular income	12 (15.4)	45 (16.5)	1.0 (0.4-1.9)	0.7 (0.3-2.1)
Education				
None or Primary	68 (87.2)	239 (87.9)	1.0	
Secondary and above	10 (12.8)	33 (12.1)	1.3 (0.5-3.0)	0.8 (0.5-1.1)
Parity				
1	38 (48.7)	85 (31.3)	2.5 (1.4-4.3)	3.2 (1.2–5.8)
2-4	27 (34.6)	149 (54.8)	1.0	
≥ 5	13 (16.7)	38 (13.9)	1.9 (0.9-4.0)	0.7 (0.3-1.3)
Prior stillbirth				
No	64 (82.1)	226 (83.1)	1.0	
Yes	14 (17.9)	46 (16.9)	1.1 (0.5-2.0)	0.8 (0.5-1.4)
Antenatal attendance				
< 4 times	3 (3.8)	216 (79.4)	1.0	1.0
≥ 4 times	75 (96.2)	56 (20.6)	1.9 (1.7-2.4)	1.3 (0.8-2.1)
Personnel checked during ANC				
Health professional	22 (28.2)	125 (46.0)	1.0	1.0
Community health worker	56 (71.8)	147 (54.0)	1.4 (1.2-1.9)	2.2 (1.3-3.7)
Knowledge of at least 2 danger signs during pregnancy				
Yes	41 (52.6)	70 (25.7)	3.1 (2.2-4.6)	2.8 (1.7-3.9)
No	37 (47.4)	202 (74.3)	1.0	1.0
Knowledge of at least 2 danger signs during childbirth				
Yes	31 (39.7)	27 (9.9)	2.3 (1.1-4.6)	1.6 (0.8-2.7)
No	47 (60.3)	245 (90.1)	1.0	
Knowledge of at least 2 danger signs during postpartum				
Yes	16 (20.5)	38 (14.0)	1.5 (0.8-2.8)	0.8 (0.5-1.4)
No	62 (79.5)	234 (86.0)	1.0	

*CI* confidence interval, *OR* odds ratio

^a^Any 3 of 4 steps: identified a skilled birth attendant, identified a health facility, arranged for transport and saved money for emergency

^b^Adjusted for all the independent variables indicated in the table

^c^Single, divorced and widowed

## Discussion

Our findings show that involving community health workers in antenatal care, as well as counseling on danger signs during pregnancy may be two effective strategies to promote birth preparedness. Although factors such as advanced maternal age, higher education, better antenatal care attendance and occupation of a woman or her partner were previously found to be associated with increased BP/CR in other studies [12, 15, 25], this was not the case in our population.

Similar to other settings, a high proportion of women reported to have received advice on BP/CR [13, 18, 19]. This may be explained by the wide availability of community health workers throughout Rwanda. Community health workers engage women and their families into formulating birth plans on a one-to-one basis prior to childbirth [26]. Still, a number of women do miss out on BP/CR advice, even if they attend antenatal care. Moreover, a considerable number of women had not followed the advice they were given, perhaps due to poor understanding of what the components of BP/CR actually entail, or to poor delivery of the messages. This finding stresses the importance of improved training for health providers on how to better communicate BP/CR-related messages and the need to address additional barriers to the uptake of BP/CR.

There were marked differences with regard to how frequent various danger signs were mentioned. In line with previous reports by others, vaginal bleeding during pregnancy, childbirth and postpartum was the most commonly reported key danger sign [16, 18]. On the contrary, prolonged labor, which is another leading cause of maternal deaths in Rwanda [22, 23] was mentioned by only few women in this study.

Our findings indicated low levels of knowledge of danger signs and birth preparedness respectively, lower than in other low-income countries [14, 18]. This may be due to our facility-based rather than community-based study setting. In addition, we applied the criterion of three out of four BP/CR components for being ‘well prepared’, where another study applied three out of five [14]. Nevertheless, the underlying principles and methods used to study BP/CR are the same.

Nulliparous women were better prepared than multiparous women, perhaps due to the misconception that after the first pregnancy BP/CR may not be required anymore. This is an indication that the frequency or quality of BP/CR messages given to multiparous women may be reduced, although these should clearly aim to also target multiparous women.

Women who knew at least two key danger signs were found more likely to be well prepared, which is similar to previous studies [12, 16, 18]. This illustrates that knowing danger signs may be an essential step towards behavioral change. This opens up possibilities for a number of potential interventions, such as the need for community-based health promotion programs and health promotion efforts at the facility in all stages of a woman’s reproductive life [27]. In addition, BP/CR requires that health services are equipped to meet the increased demand for care [28, 29].

Women who had seen community health workers had better outcomes with regard to BP/CR [26, 30]. This may be explained by the high level of community recognition for community health workers in Rwanda [26]. Therefore, in general, and particularly in settings where other health workers are scarce, community health workers should receive appropriate recognition and support [26, 31, 32].

The strength of this study is that the interview took place shortly after birth, minimizing recall bias. The fact that these women were referred for complications makes for a selected study population and it is difficult to infer our results to the general pregnant population. Moreover, some women may recall or provide information about BP/CR selectively, depending on their experience during birth or pregnancy outcome.

Nevertheless, we believe our study provides relevant information on possible opportunities to improve BP/CR. The fact that Rwanda is a densely-populated country with relatively widespread availability of health facilities (most women live less than an hour’s travel away from a facility), combined with increasing government investment in community-based health programs, performance-based financing, innovative community health insurance and SMS-based alert systems are all reasons why better implementation of BP/CR has the potential to lead to considerable improvements in pregnancy outcome in Rwanda [21, 33, 34].

## Conclusions

This study revealed low levels of knowledge of obstetric danger signs and low levels of birth preparedness among women referred to a Rwandan hospital. Prenatal advice by community health workers and knowledge of danger signs during pregnancy are associated with being better prepared for birth. Investments in health promotion with regard to BP/CR, at all stages of a woman’s reproductive life, and with support from community health workers are much needed. We recommend a review of the quality and methods of antenatal care education, including an evaluation of how multiparous women are also to benefit from such education, in order to improve the effectiveness of BP/CR.

## Additional file

Additional file 1: Consent form and questionnaire. Consent form as used in the study and questionnaire adapted from the ‘Safe Motherhood questionnaire’, as developed by the Maternal Neonatal Program of JHPIEGO, an affiliate of John Hopkins University. (DOCX 63 kb)

## Abbreviations

aOR: Adjusted odds ratio
BP: Birth Preparedness
CR: Complication Readiness
OR: Odds ratio
