# Supplementary material for: Birth preparedness and complication readiness among pregnant women admitted in a rural hospital in Rwanda
Source: BMC Pregnancy Childbirth. 2018 May 30;18:190. doi: 10.1186/s12884-018-1818-x (PMC5977552; doi:10.1186/s12884-018-1818-x)
Supplement: Supplementary file 1 — Consent form and questionnaire. Consent form as used in the study and questionnaire adapted from the ‘Safe Motherhood questionnaire’, as developed by the Maternal Neonatal Program of JHPIEGO, an affiliate of John Hopkins University. (DOCX 63 kb) [file 12884_2018_1818_MOESM1_ESM.docx]

**Consent form**

My name is ----------------------------------------------- (Interviewer)

I am working with a PhD student researcher from Athena Institute, VU University Amsterdam, The Netherlands. This study is entitled “Birth preparedness and complication readiness among pregnant women admitted in a rural hospital in Rwanda”. You are one of the women who have been selected to participate in this study. Therefore, we kindly request you to participate in this study and provide the information required. If you agree to participate in this study, you will be asked to participate in an interview which may last approximately 30-45 minutes. Your participation is voluntary and you can withdraw at any time, without giving any reason and this will not restrict you from obtaining the required medical care.

Your responses will be kept confidential and will only be used for this research purpose and only the members of the research team will have access to the data. The results of the study will be presented in publications and presentations without identifying you personally thus maintaining your confidentiality.

For any information or questions feel free to contact my supervisor Prof. dr. Jos.Van Roosmalen on +31610303726. For any questions or issues regarding your rights in this study, please contact Dr Jean Baptiste MAZARATI, the chairman of the Rwanda National Ethical Council, on Telephone number +250788309807.

Upon completion of the study, all of the data and consent forms will be shredded.

AT this time, do you want to ask me anything about the purpose or content of this interview?

Name of respondent: _________________ Signature ____________ Date: _______________

Name of interviewer: ________________Signature: _____________________

**Questionnaire**

**SECTION 1. 0 SOCIODEMOGRAPHIC INFORMATION**

| **Q. #** | QUESTIONS | | | ALTERNATIVE RESPONSES | | | | | | CODE | SKIP |
| --- | --- | --- | --- | --- | --- | --- | --- | --- | --- | --- | --- |
| 1.1 | What is your age? | | | **________**Year | | | | | |  |  |
| 1.2 | What is your current marital status? | | | 1. Married/ in Union/ Co-habiting 2. Widowed 3. Divorced 4. Separated 5. Single | | | | | |  |  |
| 1.3 | To which religion do you belong? | | | 1. Christians 2. Muslim 3. Other (specify)___________ | | | | | |  |  |
| 1.4 | Where is your place of residence (district) ? | | | 1. Musanze 2. Rubavu 3. Nyabihu 4. Burera 5. Other (specify)…………….. | | | | | |  |  |
| 1.5 | What is the highest educational level you completed? | | | 1. None 2. Stopped in primary 3. Completed primary 4. Secondary lower and above | | | | | |  |  |
| 1.6 | What is your occupation? | | | 1. None/Housewife 2. Informal employee 3. Formal/Salaried employee 4. Other (specify)………………. | | | | | |  |  |
| 1.7 | Do you have medical insurance | | | 1. Mutuel 2. RAMA/MMI 3. Other (specify)………………. 4. None | | | | | |  |  |
|  | **If** Married/ in Union/ Co-habiting | | |  | | | | | |  |  |
| 1.8 | Husband’s age (years) | | | ___________years. | | | | | |  |  |
| 1.9 | What is your husband’s highest educational level completed? | | | 1. None 2. Stopped in primary 3. Completed primary 4. Secondary lower and above | | | | | |  |  |
| 1.10 | What is your husband’s current occupation? | | | 1. Informal employee 2. Formal/Salaries employee 3. Private employee 4. Merchant 5. Other specify……… | | | | | |  |  |
| 1.11 | Monthly income of your husband. | | | ______________rfw | | | | | |  |  |
| 1.12 | Family size | | | ______________ | | | | | |  |  |
| 1.13 | What is walking distance to nearby health facility (Hours) | | | 1. < 1 2. 1-2 3. > 2 | | | | | |  |  |
| 1.14 | Who is the decision maker for health service seeking during pregnancy, delivery and postpartum period? | | | 1. Self 2. Husband 3. Community health worker 4. Other(specify)____________ | | | | | |  |  |
| 1.15 | Which Mass media do you access in your house? (more than one option is possible) | | | 1. Radio 2. Television 3. Other specify ______ | | | | | |  |  |
| 1.16 | Does any member of your house hold have a Telephone/Mobile Phone? | | | 1. Yes 2. No | | | | | |  |  |
| **SECTION 2.0: OBSTETRIC INFORMATION.** | | | | | | | | | | | |
| **Q. #** | QUESTIONS | | ALTERNATIVE RESPONSES | | | | | | | CODE | SKIP |
| 2.1 | How old were you in your first pregnancy? | | ___________years | | | | | | |  |  |
| 2.2 | How many times you became pregnant in your life? | | 1. 1 2. 2-3 3. 4 4. > 5 | | | | | | |  |  |
| 2.3 | According to your birth order where does this birth belongs? | | 1. First 2. Second 3. Third 4. Fourth and above | | | | | | |  |  |
| 2.4 | What were the outcomes of the pregnancies? | | 1. Total lives birth……. 2. Abortion………. 3. Still birth……… 4. Others (specify)….. | | | | | | |  |  |
| 2.5 | Did you attend ANC during this pregnancy | | 1. Yes 2. No 3. Does not know | | | | | | |  |  |
| 2.6 | If yes, how many times did you attend ANC | | 1. 1 2. 2-3 3. > 4 | | | | | | |  |  |
| 2.7 | At how many weeks /months of pregnancy you start ANC? | | ___________weeks/months. | | | | | | |  |  |
| 2.8 | Which personnel checked first during your first ANC | | 1. Doctor 2. Nurse/Midwife 3. Community health worker 4. Other (Specify)…………… | | | | | | |  |  |
| 2.9 | Have you ever heard the term “birth preparedness? | | 1. Yes 2. No | | | | | | |  | Q2.11 |
| 2.10 | If yes; From whom did you get the information? **(more than one answer is possible)** | | 1. Health professional 2. CHW 3. Mother/Mother-in-law and relatives 4. Media: TV or Radio 5. Other(specify)_______________ | | | | | | |  |  |
| 2.11 | In your opinion, does a woman need preparation for birth? | | 1. Yes 2. No | | | | | | |  | Q3.1 |
| 2.12 | In your opinion, what are some things a woman can do to prepare for birth?  (**more than one answer is possible)** | | 1. Identify place of delivery 2. Save money 3. Prepare essential items for clean delivery & post partum period 4. Identify SBA 5. Being aware of the signs of an emergency & the need to act immediately 6. Designating decision maker on her 7. Arranging a way to communicate with a source of help 8. Arranging emergency funds 9. Identify a mode of transportation 10. Other (specify) ------------------------------ | | | | | | |  |  |
| **SECTION 3.0፡ PRACTICES OF RESPONDENTS ON BIRTH PREPAREDNESS AND COMPLICATION READINESS** | | | | | | | | | | | |
| **3.1** | Did you identify place of delivery? | | 1. Yes 2. No | | | | | | |  | Q3.3 |
| 3.2 | If yes, Where was the place of delivery you planned? | | 1. Government hospital 2. Health center 3. Dispensary 4. Home 5. Others specify | | | | | | |  |  |
| 3.3 | Where did you prefer to deliver from here? | | Government hospital | | | | | | |  | Q3.5 |
| 3.4 | If **home**, Why did you prefer to deliver at home?  (**More than one response is possible**) | | 1. Too much cost of HFs 2. Facility too far 3. Poor quality service of HFs 4. No female provider at HFs 5. Husband will not allow 6. Need to be with relatives 7. Presence of TBAs 8. Labor was smooth and short 9. Previous home delivery was normal 10. Lack of accompanies 11. I was told my pregnancy is normal 12. Lack of transport 13. Others specify………………………….. | | | | | | |  |  |
| 3.5 | If HFs, Why did you prefer to deliver in health facilities? (more than one answer is possible) | 1. HF was near to me 2. Need better service 3. Previous better outcome with delivering at HF 4. I was told to deliver at health facilities 5. Difficult labor 6. Bad outcome with previous delivery 7. HF delivery is always needed 8. Other……………………………………………….. | | | | | | | |  |  |
| 3.6 | What was the mode of delivery? | 1. Spontaneous vaginal delivery 2. Cesarean section 3. Instrumental delivery 4. Other specify……………………………….. | | | | | | | |  |  |
| 3.7 | Did you plan skilled assistant during delivery? | | | | | | | | 1. Yes 2. No |  | Q3.9 |
| 3.8 | If yes, Whom had you planned to assist you? | | 1. Medical doctor 2. Midwife/Nurse 3. Other (specify) | | | | | | |  |  |
| 3.9 | Who was your birth attendant? | | 1. Health Professional 2. Community health worker 3. Mother/Mother-in law/Relatives 4. If other, explain ----- | | | | | | |  |  |
| 3.10 | Had you planned to save money for obstetric emergency? | | 1. Yes 2. No | | | | | | |  |  |
| 3.11 | Had you saved money for obstetric emergency? | | 1. Yes 2. No | | | | | | |  |  |
| 3.12 | Had you planned a mode of transport to place of delivery during emergency? | | | | 1. Yes 2. No | | | | |  | Q3.13 |
| 3.13 | If yes, What was a mode of transport you had planned?  (ask those planned for emergency transport) | | 1. On foot 2. Motorcycle 3. Carried by other people 4. Car 5. Ambulance 6. Others specify……… | | | | | | |  |  |
| 3.14 | Can you detect early signs of an Emergence? | | 1. Yes 2. No | | | | | | |  |  |
| 3.15 | Did you encounter any health problems during labor, delivery and immediately after birth? | | | | | | | 1. Yes 2. No | |  | Q3.19 |
| 3.16 | If yes, what were the problems? (more than one answer is possible) | | 1. Excessive vaginal bleeding 2. Prolonged labor (> 12hrs) 3. Retained placenta (>1hrs) 4. Abnormal fetal lie 5. Fetal death 6. Early rupture of membrane 7. Loss of consciousness 8. Other specify……………………………… | | | | | | |  |  |
| 3.17 | Were you referred to HF further? (ask those who faced the problem) | | | | | | | | 1. Yes 2. No |  |  |
| 3.18 | If you were referred to HF, who accompanied you to HF(ask referred) | | 1. Husband 2. Mother/Mother-in-law/Relatives 3. CHWs 4. Alone 5. Others specify…………………………. | | | | | | |  |  |
| 3.19 | If you were referred to health facility, what mode of transport did you use to reach to the health facility? (ask referred) | | 1. On foot 2. Motorcycle 3. Carried by other people 4. Car 5. Ambulance 6. Others specify……… | | | | | | |  |  |
| **SECTION 4.0: - AWARENESS OF OBSTETRIC DANGER SIGNS** | | | | | | | | | | | |
| Q.# | QUESTIONS | | ALTERNATIVE RESPONSES | | | | | | | CODE | SKIP |
| 4.1 | Are there any obstetric danger signs that can occur during pregnancy, labor and postpartum? | | | | | | 1. Yes 2. No | | |  | Q4.3 |
| 4.2 | If yes, from where did you hear these danger signs? (more than one answer is possible) | | 1. Health professionals 2. Community health worker 3. Radio 4. Television 5. Read from books 6. Other (specify)………………………… | | | | | | |  |  |
| 4.3 | Are there any danger signs that can occur during pregnancy? | | 1. Yes 2. No | | | | | | |  | Q4.6 |
| 4.4 | What are these danger signs?  (Wait them to spontaneously mention some or not) more than one answer is possible) | | 1. Vaginal bleeding 2. Severe headache 3. Blurred vision 4. Convulsions 5. Swollen hands/face*.* 6. High fever 7. Loss of consciousness 8. Difficulty breathing 9. Severe weakness 10. Severe abdominal pain 11. Accelerated/reduced fetal movement 12. Water breaks without labor 13. Other (Specify)---------------------- | | | | | | |  |  |
| 4.5 | In your opinion, could women die from [this problem] any of these problems? | | 1. Yes 2. No   98. Don’t know | | | | | | |  |  |
| 4.6 | Are there any danger signs that can occur during labor or child birth? | | | | | 1. Yes 2. No | | | |  | Q4.9 |
| 4.7 | What are these danger signs?  (Wait them to spontaneously mention some or not) more than one answer is possible) | | 1. Severe vaginal bleeding 2. Severe headache 3. Convulsions 4. High fever 5. Loss of consciousness 6. Labor lasting>12hours 7. Placenta not delivered 30 minutes after delivery 8. Other (Specify) | | | | | | |  |  |
| 4.8 | In your opinion, could women die from [this problem] any of these problems? | | 1. Yes 2. No   98. Don’t know | | | | | | |  |  |
| 4.9 | Are there any danger signs that can occur during postpartum period? | | | 1. Yes 2. No | | | | | |  |  |
| 4.10 | What are danger signs that can occur during postpartum?  (Wait them to spontaneously mention some or not) more than one answer is possible | | 1. Severe vaginal bleeding 2. Severe headache 3. Blurred vision. 4. Convulsions 5. Swollen hands/face 6. High fever 7. Loss of consciousness. 8. Difficulty breathing. 9. Severe weakness 10. Malodorous vaginal discharge 11. Others (specify)__________ | | | | | | |  |  |
| 4.11 | In your opinion, could women die from [this problem] any of these problems? | | 1. Yes 2. No   98. Don’t know | | | | | | |  |  |

Thank you for your time and valuable information you gave us. Do you have any question that I can address for you?

………………………………………………………………………………………………………………………………………………………………………………………………………………………………………………………………………………………………………………………
